# The controversial role of the vagus nerve in mediating ghrelin's actions: gut feelings and beyond

**DOI:** 10.1016/j.ibneur.2022.03.003

**Published:** 2022-03-12

**Authors:** Mario Perelló, María P. Cornejo, Pablo N. De Francesco, Gimena Fernandez, Laurent Gautron, Lesly S. Valdivia

**Affiliations:** a Laboratory of Neurophysiology of the Multidisciplinary Institute of Cell Biology [IMBICE, Argentine Research Council (CONICET) and Scientific Research Commission, Province of Buenos Aires (CIC-PBA), National University of La Plata], La Plata, Buenos Aires, Argentina; b Department of Surgical Sciences, Functional Pharmacology and Neuroscience, University of Uppsala, Uppsala, Sweden; c Center for Hypothalamic Research, Department of Internal Medicine, UT Southwestern Medical Center, Dallas, TX, USA

**Keywords:** AgRP, agouti-related protein, Amb, nucleus ambiguous, AP, area postrema, ARH, hypothalamic arcuate nucleus, CART, cocaine, and amphetamine-regulated transcript, CB1, cannabinoid receptor type 1, CCK, cholecystokinin, DMNV, dorsal motor nucleus of the vagus, DVC, dorsal vagal complex, ERK, extracellular signal-regulated kinases, GABA, gamma aminobutyric acid, GH, growth hormone, GHSR, growth hormone secretagogue receptor, GI, gastrointestinal, GLP 1, glucagon-like peptide 1, ICV, intracerebroventricularly, IP, intraperitoneally, ISH, in situ hybridization, IV, intravenously, JG, jugular ganglion, LEAP2, liver-expressed antimicrobial peptide 2, MMC, migrating motor complex, NG, nodose ganglion, NTS, nucleus of the solitary tract, PCR, polymerase chain reaction, SC, subcutaneously, TRPV1, transient receptor potential vanilloid receptor 1, Ghrelin receptor, GHSR, Nodose ganglion, Dorsal vagal complex, Autonomic nervous system

## Abstract

Ghrelin is a stomach-derived peptide hormone that acts via the growth hormone secretagogue receptor (GHSR) and displays a plethora of neuroendocrine, metabolic, autonomic and behavioral actions. It has been proposed that some actions of ghrelin are exerted via the vagus nerve, which provides a bidirectional communication between the central nervous system and peripheral systems. The vagus nerve comprises sensory fibers, which originate from neurons of the nodose and jugular ganglia, and motor fibers, which originate from neurons of the medulla. Many anatomical studies have mapped GHSR expression in vagal sensory or motor neurons. Also, numerous functional studies investigated the role of the vagus nerve mediating specific actions of ghrelin. Here, we critically review the topic and discuss the available evidence supporting, or not, a role for the vagus nerve mediating some specific actions of ghrelin. We conclude that studies using rats have provided the most congruent evidence indicating that the vagus nerve mediates some actions of ghrelin on the digestive and cardiovascular systems, whereas studies in mice resulted in conflicting observations. Even considering exclusively studies performed in rats, the putative role of the vagus nerve in mediating the orexigenic and growth hormone (GH) secretagogue properties of ghrelin remains debated. In humans, studies are still insufficient to draw definitive conclusions regarding the role of the vagus nerve mediating most of the actions of ghrelin. Thus, the extent to which the vagus nerve mediates ghrelin actions, particularly in humans, is still uncertain and likely one of the most intriguing unsolved aspects of the field.

## 1 Introduction

Ghrelin is a gastrointestinal (GI) tract-derived hormone that displays key neuroendocrine, metabolic, autonomic and behavioral effects. Most GI hormones, including cholecystokinin (CCK) and glucagon-like peptide 1 (GLP-1), act on sensory neurons of the vagus nerve, which is recognized as a critical regulator of many aspects of physiology, such as food intake, cardiovascular functions, GI motility or digestion. In line with this general concept, it has been proposed that ghrelin also exerts some of its effects, at least in part, via vagally-mediated mechanisms. However, and despite many studies published on the topic, the evidence for the role of the vagus nerve mediating some actions of ghrelin is still a matter of debate. Here, we critically review the available evidence supporting, or not, the notion that the vagus nerve mediates the actions of ghrelin.

## 2 Ghrelin

Ghrelin is an octanoylated peptide hormone mainly released from endocrine cells of the stomach (Kojima et al., 1999). The octanoylation of ghrelin is mandatory for its bioactivity. Ghrelin acts via GHSR, a G protein-coupled receptor highly expressed in the brain, the pituitary and some peripheral organs, including the pancreas (Guan et al., 1997, Howard et al., 1996, Zigman et al., 2006). Canonically, ghrelin-evoked GHSR activity recruits Gq protein signaling that triggers activation of extracellular signal-regulated kinases (ERK) and increment of intracellular calcium that, in turn, up-regulates a variety of calcium-sensitive pathways (Yin et al., 2014).

Plasma ghrelin levels depend on the short-term feeding status, rising before set meals and decreasing post-prandially (Cummings et al., 2001), as well as on the long-term energy balance, being lower in individuals with obesity and higher in energy deficit conditions, such as fasting (Cummings et al., 2002, Müller et al., 2015, Tschöp et al., 2001b). Plasma ghrelin levels are also elevated in individuals with anorexia nervosa, cancer-associated cachexia and chronic obstructive pulmonary disease, among other health conditions with severe energy deficits (Müller et al., 2015). The actions of ghrelin become more evident under conditions of severe energy deficit. For instance, mice lacking ghrelin and exposed to calorie restriction fail to appropriately rise plasma GH levels, display severe hypoglycemia and become moribund (Goldstein et al., 2011, Zhao et al., 2010). Also, GHSR-deficient mice display a reduced compensatory hyperphagia following a prolonged fasting (Fernandez et al., 2018). The cardinal functions of ghrelin are also revealed after its systemic administration. Ghrelin treatment increases GH and glucocorticoid levels in plasma (Arvat et al., 2001, Cabral et al., 2012, Kojima et al., 1999, Wren et al., 2000). Ghrelin administration also rapidly increases food intake and promotes body weight gain (Nakazato et al., 2001, Tschöp et al., 2001a, Wren et al., 2001). Moreover, ghrelin treatment activates mechanisms that raise glycemia, which include increments of GH and glucocorticoid secretion as well as inhibition and enhancement, respectively, of insulin and glucagon secretion from pancreas (Mani et al., 2019). Ghrelin also acts at the GI tract stimulating motility, gastric emptying, gastric acid secretion and pancreatic excretion (Greenwood-Van Meerveld et al., 2011, Masuda et al., 2000, Sanger et al., 2017). In healthy subjects, ghrelin treatment reduces blood pressure and heart rate (Nagaya et al., 2001). Importantly, intracerebroventricularly (ICV)-injected ghrelin displays a variety of actions in rodents, such as stimulation of locomotor activity, enhancement of reward-related behaviors, modulation of stress and anxiety-like behaviors, improvement of learning and memory consolidation, among others (Cornejo et al., 2021a). The extent to which plasma ghrelin can reach brain areas expressing GHSR is currently controversial. Studies in mice, for instance, showed that plasma ghrelin displays a restricted accessibility to the brain that is limited to brain areas containing or located nearby fenestrated capillaries —e.g., the hypothalamic arcuate nucleus (ARH) or the area postrema (AP)— (Perello et al., 2019). In rats, however, some evidence showed that circulating ghrelin is able to act in some deep brain areas and display some effects (Hsu et al., 2018, Skibicka et al., 2011). Regardless of the apparent discrepancy between the wide distribution of GHSR in the brain and a presumably limited accessibility of ghrelin into the brain (for review see Abizaid et al., 2006; Cornejo et al., 2021a), it seems evident that some key physiological effects of ghrelin would still mainly depend on the targets that can indeed sense the endogenous fluctuations of its plasma levels.

## 3 The vagus nerve

The vagus nerve, also known as the pneumogastric or tenth cranial nerve, is the most complex of the cranial nerves. The vagus nerve carries both vagal efferent and sensory afferent fibers (Câmara and Griessenauer, 2015). Efferent fibers originate from the dorsal motor nucleus of the vagus (DMNV), responsible of the parasympathetic motor efferents especially to the GI tract, or the nucleus ambiguus (Amb), which generates branchial and parasympathetic motor efferents that innervate the heart (Ruffoli et al., 2011). Functionally, vagal efferents are grouped as either parasympathetic motor efferents, which provide preganglionic cholinergic innervation to smooth muscle and glands of the pharynx, larynx, thorax and GI tract, or branchial motor efferents that innervate striated muscles in the pharynx, larynx and esophagus. Depending on their conduction velocity, vagal fibers are classified from faster to slower into A-fibers that are myelinated and include visceral afferents and branchial motor efferents; B-fibers that are also myelinated and include parasympathetic motor efferents; and C-fibers that are non-myelinated and include visceral afferents (Ruffoli et al., 2011).

Vagal sensory afferents originate from pseudo-unipolar neurons located in the jugular ganglion (JG) and the nodose ganglion (NG). Sensory afferents represent ~80% of vagal fibers and transmit information from the heart, the respiratory tract and most of the GI tract. Sensory afferents can be grouped into: general somatic afferents, which originate from the JG and carry touch, pressure, pain and temperature sensations, general visceral afferents, which originate from the NG and convey mechano- and chemosensory sensations, and special visceral afferents, which also originate from the NG and provide gustatory inputs (Câmara and Griessenauer, 2015, Ruffoli et al., 2011). Most taste and primary afferents from visceral organs target the nucleus of the solitary tract (NTS), whereas afferent information from orofacial regions targets the spinal trigeminal nucleus (Ruffoli et al., 2011). The NTS receives and integrates afferent vagal information and then innervates a wide variety of brain areas, including the DMNV, which, together with the AP and the NTS forms the dorsal vagal complex (DVC).

At neuroanatomical level, the vagus nerve egresses on each side of the medulla and forms the left and right vagal nerves. As they exit the cranium, vagal fibers pass through the JG and NG (Berthoud and Neuhuber, 2000, Câmara and Griessenauer, 2015). The auricular and meningeal branches of the vagal nerves emerge from the JG, whereas the pharyngeal and superior laryngeal branches exit from the NG. The main cervical trunks emerge distal to the NG and produce the cervical cardiac branches and the recurrent laryngeal nerves. Upon reaching the lung roots, the branches of the vagal nerve give rise to the esophageal plexus as well as to the pulmonary branches and cardiac branches that, in turn, innervate postganglionic neurons. The left and right vagus nerves then enter the abdominal cavity becoming in the subdiaphragmatic ventral and dorsal vagal trunks, respectively. In several species, the ventral and dorsal abdominal vagal trunks carry fibers from both the left and right vagus nerves due to partial mixing at the esophageal plexus (Berthoud and Neuhuber, 2000, Câmara and Griessenauer, 2015). The ventral trunk divides into the ventral gastric branch, the ventral celiac branch, and the common hepatic branch, which further supplies liver, gallbladder, duodenum, pylorus and pancreas (Berthoud and Neuhuber, 2000). The dorsal trunk innervates the dorsal face of the stomach, the proximal duodenum, and gives off the dorsal celiac branch, which is joined by the ventral celiac branch, providing innervation to several target organs, including neurons in the pancreas (Tao et al., 2021), mesenteric ganglion (Berthoud and Powley, 1993) and enteric plexuses (Gautron et al., 2013).

The vagus nerve modulates cardiovascular function via its parasympathetic efferents, depressing heart rate and contractility, and by conveying inputs from the aortic arch baroreceptors to the cardioinhibitory center for the baroreflex (Min et al., 2019). Also, vagal parasympathetic fibers innervating chemoreceptors in the carotid body decrease respiration rate and airway tone (Zera et al., 2019). The vagus nerve integrates the GI function in the DVC and potently regulates its activity via vago-vagal reflexes (Travagli et al., 2006). In particular, vagal afferents from the GI tract carry mechanical, osmo- and chemosensory information to the caudal NTS, which modulates parasympathetic efferents from the DMNV resulting in increased GI motility (including gastric emptying, increased peristalsis, relaxation of sphincters), gastric secretion and pancreatic enzyme production (Dockray, 2013).

## 4 Evidences unmasking direct targets of ghrelin at the vagus nerve

### 4.1 GHSR in sensory neurons

A seminal study by Date and colleagues detected GHSR mRNA in the rat NG using reverse transcription polymerase chain reaction (PCR) and *in situ* hybridization (ISH) (Date et al., 2002). Subsequent studies corroborated GHSR expression in the rat NG using the same techniques (Burdyga et al., 2006, Davis et al., 2020, Paulino et al., 2009, Sakata et al., 2003, Sato et al., 2007) and showed that ~70% of NG neurons contain GHSR mRNA detected by FISH (Davis et al., 2020). The combined use of ISH and fluorescent tracing indicated that GHSR-expressing cells of the rat NG innervate the stomach (Sakata et al., 2003). In rats, GHSR mRNA levels in the NG were found increased during fasting (Sato et al., 2007), although other study could not confirm such observation (Burdyga et al., 2006). GHSR mRNA was also detected in the mouse NG (Davis et al., 2020, Kentish et al., 2012, Page et al., 2007, Zhang et al., 2020). Particularly, GHSR mRNA was found in mouse NG neurons innervating the stomach that, in turn, expressed GLP-1 receptor, transient receptor potential vanilloid receptor 1 (TRPV1) and cannabinoid receptor type 1 (CB1), and such GHSR expression increased in high-fat diet-fed mice and in fasted mice (Christie et al., 2020, Kentish et al., 2012, Zhang et al., 2020). Other studies, however, failed to detect GHSR mRNA in mouse NG using real time PCR and single-cell RNA sequencing (Egerod et al., 2018, Kupari et al., 2019). A recent study using multiplex ISH showed that GHSR mRNA is undetectable in mouse NG neurons, and detectable in 3% of JG neurons (Bob-Manuel and Gautron, 2021). In line with the notion that ghrelin is not produced in the nervous system (Cabral et al., 2017), the mRNAs for ghrelin or the enzyme that acylates ghrelin were absent in NG and JG neurons (Kupari et al., 2019). Thus, convincing evidence indicates that GHSR is expressed in rat vagal sensory neurons. In contrast, the extent to which GHSR is expressed in mouse vagal sensory neurons is controversial. Importantly, the NG and the JG are almost fused in rodents; thus, it is likely that contaminations of NG samples with JG tissue may explain some discrepancies found in gene expression analysis, or in studies investigating cultured vagal sensory cells (see below).

Immunohistochemical analysis indicated that GHSR is present in the rat NG and that ~70% of GHSR-immunoreactive cells express CCK-A receptor (Burdyga et al., 2006, Date et al., 2005). GHSR-immunoreactive cells of the NG were also shown to express CB1, melanin-concentrating hormone receptor 1, TRPV1 and tyrosine hydroxylase (Burdyga et al., 2006, Date et al., 2005, Mano-Otagiri et al., 2010), and GHSR immunoreactive fibers were found in the stomach and colon of rats and humans (Dass et al., 2003). Of note, three of the above referred studies validated the specificity of the tested antibodies *in vitro* (Dass et al., 2003, Date et al., 2005, Mano-Otagiri et al., 2010) but not *in vivo*, which is particularly problematic for antibodies against G-protein coupled receptors (Michel et al., 2009). Strikingly, faintly stained GHSR-immunoreactive glial cells were found in primary cell cultures of rat NG, using a non-validated commercial antibody (Avau et al., 2013). Binding studies showed that I^125^-ghrelin labeled ~40% of NG neurons and that labeling accumulated in the proximal segment after ligation of vagal segments in rats, suggesting that GHSR is transported towards the terminals (Date et al., 2002). Likewise, I^125^-ghrelin labeling was shown to accumulate in the proximal segments after ligation of vagal segments in mice (Zhang et al., 2020).

Some functional evidence also indicates that ghrelin acts in vagal sensory neurons. In cultured rat NG neurons, ghrelin abolished CCK-induced cocaine- and amphetamine-regulated transcript (CART) expression and the mobilization to the cell nucleus of some transcription factors (de Lartigue et al., 2010, de Lartigue et al., 2007). Also, ghrelin increased ERK1/2 phosphorylation in cultured rat NG neurons through Gi protein coupling (Grabauskas et al., 2015). *In vivo*, subcutaneously (SC)-injected ghrelin increased ERK2 phosphorylation in mouse NG neurons, an effect abolished by high-fat diet feeding (Naznin et al., 2015), although such observation does not indicate a direct action of ghrelin. One study found that ghrelin increased intracellular calcium in ~3% of cultured rat vagal sensory neurons (Avau et al., 2013). In cultured mouse NG neurons, ghrelin did not affect basal or CCK-induced increase of intracellular calcium, but inhibited insulin-induced increase of intracellular calcium (Iwasaki et al., 2015). Likewise, other study found that ghrelin hyperpolarizes cultured mouse NG cells but does not affect intracellular calcium levels (Zhang et al., 2020). Of note, ghrelin treatment increased vagal fibers activity in an *in situ* model of decorticated artificially perfused rats, in which the infused solutions do not reach NG cell bodies (Davis et al., 2020) indicating that vagal sensory neurons could sense circulating ghrelin not only at the cell body but also at the terminals.

### 4.2 GHSR in efferent neurons of the vagus nerve

GHSR mRNA is present in the DMNV of mice and rats, as evidenced by reverse transcription PCR (Zhang et al., 2004), ISH (Bron et al., 2013, Scott et al., 2012, Zigman et al., 2006, Zigman et al., 2005), and transgenic mice that express enhanced green fluorescent protein under the GHSR promoter (GHSR-eGFP mice, (Cornejo et al., 2018; Mani et al., 2014)). GHSR immunoreactive cells were also detected in the DMNV, although the specificity of the tested antibodies was not properly assessed in those studies (Lin et al., 2004, Zhang et al., 2004). ICV-injected fluorescent ghrelin also labeled the DMNV (Cabral et al., 2013). Functional studies indicated that DMNV responds to ghrelin. In particular, ICV- or intravenously (IV)-injected ghrelin in rats increased the expression of the marker of neuronal activation c-Fos in the DMNV (Date et al., 2001, Li et al., 2006). Moreover, oral administration of ghrelin analogs increased c-Fos in the DMNV of shrews (Tu et al., 2020), and ICV-injected ghrelin increased phosphorylation of the mammalian target of rapamycin in the DMNV of rats (Zhang et al., 2013). Importantly, GHSR signaling does not specifically recruit any of the referred signaling proteins and, consequently, the referred functional studies may involve indirect actions of ghrelin on the DMNV. Electrophysiological recordings in rat brain slices indicated that ghrelin increases the frequency but does not affect the amplitude of spontaneous glutamatergic currents of DMNV neurons, an indication that such effect is mainly indirect (Besecker et al., 2018, Swartz et al., 2014). GHSR mRNA is present in the Amb of rats and mice, as revealed by ISH (Bron et al., 2013, Scott et al., 2012, Zigman et al., 2006), and fluorescent cells were observed in the Amb of the GHSR-eGFP mice (Mani et al., 2014). Notably, the DMNV and the Amb are protected by the blood-brain barrier (Maolood and Meister, 2009), and were not reached by SC-injected fluorescent ghrelin (Cabral et al., 2014), indicating that cell bodies located in these brain nuclei cannot sense plasma ghrelin levels in mice. To our knowledge, there is not published neuroanatomical evidence that GHSR is present in the terminals of vagal efferents.

### 4.3 GHSR in other brain areas that modulate vagus nerve activity

GHSR is expressed in rat and mouse NTS as shown by ISH (Zigman et al., 2006, Zigman et al., 2005). GHSR-expressing neurons were also detected in the NTS of GHSR-eGFP mice (Cornejo et al., 2018, Mani et al., 2014) and ICV-injected fluorescent ghrelin labeled the NTS in mice (Cabral et al., 2014, Cabral et al., 2013, Cornejo et al., 2018). Also, ICV- or systemically-injected ghrelin increased c-Fos in the NTS of mice and rats (Cabral et al., 2017, Cabral et al., 2014, Cornejo et al., 2018, Hashimoto et al., 2007, Li et al., 2006, Takayama et al., 2007). In mice, GHSR-expressing neurons of the NTS were found segregated in two rostral clusters, which are activated in response to food intake, and a caudal cluster that include gamma aminobutyric acid (GABA)-producing neurons and is activated in response to gastric distension or malaise (Cornejo et al., 2018). Of note, GHSR-expressing neurons of the mouse NTS were non-catecholaminergic neurons, which indirectly responded to ghrelin as the hormone inhibits glutamate transmission onto them (Cui et al., 2011). In line with this observation, ICV-injected ghrelin increased c-Fos in non-catecholaminergic neurons of the rat NTS (Faulconbridge et al., 2008). As described for the DMNV and the Amb, SC-injected fluorescent ghrelin did not target the NTS (Cabral et al., 2017). To our knowledge, there are no reports indicating that GHSR is expressed in the spinal trigeminal nucleus.

The AP contains highly vascularized blood vessels devoid of a blood-brain barrier and can sense circulating factors (Maolood and Meister, 2009). GHSR is highly expressed in the rat and mouse AP as shown by ISH (Zigman et al., 2006, Zigman et al., 2005). GHSR-expressing neurons were also detected in the AP of GHSR-eGFP mice (Cabral et al., 2017, Mani et al., 2014) and fluorescent ghrelin labeled the AP in mice (Cabral et al., 2017, Cabral et al., 2014, Cabral et al., 2013). Also, ICV- or systemically-injected ghrelin increased c-Fos in the AP of mice and rats (Cabral et al., 2017, Cabral et al., 2014, Cabral et al., 2013, Hashimoto et al., 2007, Li et al., 2006, Takayama et al., 2007). In mice, most GHSR-expressing neurons of the AP were GABA neurons and increased c-Fos expression in response to fasting in a GHSR-dependent manner (Cabral et al., 2017). *In vitro*, ghrelin acted on ~40% of rat AP neurons, with half of them showing a depolarization and half showing a hyperpolarization of their membrane potential (Fry and Ferguson, 2009). Notably, IV-injected ghrelin did not increase c-Fos in the NTS of AP-ablated rats (Li et al., 2006), and GHSR-expressing neurons of the AP were shown to send ventrolateral projections towards the NTS in mice (Cabral et al., 2017). Thus, GHSR-expressing neurons of the AP likely play a key role sensing plasma ghrelin and regulating vagal circuits.

The DVC is modulated by inputs originating from upstream brain regions (Cooper et al., 2021), such as the ARH, which contain Agouti-related protein (AgRP)-expressing neurons that express high levels of GHSR and sense circulating ghrelin (Campbell et al., 2017, Perello et al., 2019). The ARH has reciprocal connections with the NTS (Wang et al., 2015). The observation that ablation of AgRP neurons increases c-Fos in the NTS suggests that they directly or indirectly inhibit the NTS (Wu et al., 2008); however, the extent to which this pathway affects vagal functions remains uncertain. Alternatively, the NTS may modulate the ARH. For instance, catecholaminergic fibers from NTS were shown to innervate the ARH and its optogenetic activation promoted food intake (Aklan et al., 2020). Similarly, calcitonin receptor-expressing fibers from the NTS were shown to innervate the ARH and its pharmacogenetic activation blocked ghrelin-induced food intake (Cheng et al., 2020). Conversely, a recent study using a combination of retrograde viral tracing strategies found no evidence of inputs from the NTS to the ARH (Tsang et al., 2020). Thus, the lack of unequivocal evidence on the extent and nature of the interconnections between the NTS and the ARH prevent us to further theorize their putative role mediating ghrelin's actions on the vagus nerve.

## 5 Studies investigating the role of the vagus nerve mediating some actions of ghrelin

The role of the vagus nerve mediating specific actions of ghrelin has been studied using different experimental strategies to vagotomize animals (Wang et al., 2020). Surgical vagotomy is a classical strategy to physically interrupt vagal fibers. Surgical vagotomy includes either total (bilateral) subdiaphragmatic vagotomy, which leads to severe hypophagia and body weight loss and requires the use of liquid diets to minimize post-vagotomy symptoms, or selective vagotomy such as gastric vagotomy that has less severe consequences (Kraly et al., 1986). Importantly, vagotomy abolishes afferent and efferent transmissions and, consequently, it does not allow to study their differential roles. Conversely, subdiaphragmatic vagal deafferentation is a surgical strategy that involves the removal of all subdiaphragmatic vagal afferents whereas it maintains around half of efferents and, consequently, has fewer side effects (Norgren and Smith, 1994, Schwartz et al., 1997). Alternatively, the perivagal exposure to the excitotoxin capsaicin is used to produce a selective degeneration of sensory neurons and fibers in specific organs (e.g., gastric branch vagotomy). Initially, it was assumed that capsaicin mainly degenerates and diminishes the sensory signaling of unmyelinated afferents (C-type fibers) expressing TRPV1 whereas preserves myelinated fibers (A- and B-type fibers) (Czaja et al., 2008, Holzer, 1998, Pingle et al., 2007, Ritter and Dinh, 1988). However, many other neuronal populations of the mouse brain, including neurons of the DVC, abundantly express TRPV1 (Cavanaugh et al., 2011), and perivagal capsaicin application has been shown to alter membrane properties and neurotransmitter responsiveness of DMNV neurons in rats (Browning et al., 2013). Consequently, studies using capsaicin-treated animals should be interpreted with caution and should ideally include alternative strategies of vagotomies and/or verification of the degree of degeneration of neurons other than sensory neurons. Finally, it is important to highlight that vagal fibers regenerate several weeks or months after surgical or chemical vagotomy in rats and mice (Czaja et al., 2008, Gallaher et al., 2011, Phillips et al., 2003, Powley et al., 2005, Ryu et al., 2010). The regeneration of vagal fibers after vagotomy, however, is neither complete nor entirely accurate, and the extent to which regenerated fibers are fully functional remains uncertain. Thus, studies in vagotomized animals need to be performed in carefully chosen time frames after animal recovery but before regeneration of vagal fibers.

The following sections discuss the studies addressing the putative role of the vagus nerve mediating specific actions of ghrelin:

### 5.1 Food intake

Asakawa and colleagues first reported that intraperitoneally (IP)-injected ghrelin does not affect food intake in mice with total subdiaphragmatic vagotomy (Asakawa et al., 2001). A subsequent study found that IV-injected ghrelin does not affect food intake in rats with total subdiaphragmatic vagotomy or in rats with capsaicin-induced or surgical gastric vagotomy (Date et al., 2002). Notably, ICV-injected ghrelin fully increased food intake in these models of vagotomized rats (Date et al., 2002). In a subsequent study, the same team found that IV-injected ghrelin does not induce feeding in rats with bilateral midbrain transections rostral to the NTS to disrupt the connections between the medulla and the rest of the brain (Date et al., 2006). Also, IV-injected ghrelin did not induce food intake in rats pretreated with α1 or β2 antagonists or with selective ablation of catecholaminergic innervations to the ARH (Date et al., 2006). Notably, ICV-injected ghrelin fully induced food intake in all three described experimental models (Date et al., 2006). Thus, it was proposed that the vagally-mediated orexigenic effect of systemically-injected ghrelin involves catecholaminergic NTS neurons that, in turn, release noradrenaline in the ARH and stimulate feeding. Another study reported that IP-injected ghrelin does not affect food intake in 16-h food deprived rats receiving perivagal treatment with capsaicin at the cervical level (Chen et al., 2005). In sharp contrast, Arnold and colleagues found that IP-injected ghrelin fully increases food intake in rats with subdiaphragmatic vagal deafferentation or total subdiaphragmatic vagotomy (Arnold et al., 2006). The reasons for the inconsistencies are uncertain. The studies that failed to detect ghrelin-induced food intake in vagotomized rats were performed at 7 (Chen et al., 2005, Date et al., 2002) or 4 (Asakawa et al., 2001) days after surgery, presumably when animals still displayed acute consequences of vagotomy. In contrast, the study that detected ghrelin-induced food intake in vagotomized rats was performed 6 weeks after deafferentation or 3 weeks after vagotomy, when rats already consumed large meals. In contrast to the other three studies, the later study verified the lesion when testing the effects of ghrelin by using functional (i.e., lack of response to the anorectic effects of CCK) and histological (i.e., lack of retrograde labeling of the DMNV after intraperitoneal injection of fluorogold) tests. A recent study reported that systemically-injected ghrelin does not affect food intake during the nocturnal phase in rats tested ~3 months after total subdiaphragmatic vagotomy (Davis et al., 2020). This study argues that conducting feeding studies in the dark cycle may have helped to unmask the requirement of the vagus for ghrelin-induced food intake since GHSR gene expression in the NG is lower during the dark cycle (Sato et al., 2007). An independent study found that ghrelin does not affect food intake in rats in which the gene expression of Kir6.2 channel subunit, a critical component of a specific potassium channel, was silenced in the NG (Grabauskas et al., 2015), although several concerns have been raised in terms of some data sets of this study (https://pubpeer.com/publications/4ACA2E2C887F2F806D1D3066B6A85A). In order to clarify the role of ghrelin in vagal afferents, a recent study performed knockdown of GHSR in the NG and found that such manipulation increased meal frequency, but not meal size nor cumulative chow intake during the dark cycle, and increased body weight (Davis et al., 2020). Unfortunately, the study did not report if ghrelin treatment affects food intake in rats with knockdown of GHSR in the NG. Thus, it remains uncertain if the orexigenic effect of ghrelin requires its direct action on vagal sensory neurons in rats. Of note, the orexigenic effect of ghrelin does not involve the AP since SC-injected ghrelin fully increases food intake in AP-lesioned rats (Gilg and Lutz, 2006). However, the administration of ghrelin into the DVC (Faulconbridge et al., 2003) or the fourth ventricle (Zhang et al., 2013) increased food intake in rats, suggesting that the action of ghrelin on vagal centers of the medulla can trigger appetite.

After the initial study by Asakawa and colleagues, few studies investigated if the orexigenic effect of ghrelin in mice involves the vagus nerve. A study found that daily SC-injected ghrelin in mice with gastrectomy, which includes total subdiaphragmatic vagotomy, partially reverses the gastrectomy-induced reduction in body weight (Dornonville de la Cour et al., 2005). Also, ghrelin did not induce food intake in mice with GHSR expression restricted to Phox2b-expressing neurons, which includes neurons of the NTS, NG, DMNV, AP, Amb and facial motor nucleus (Okada et al., 2018, Scott et al., 2012), suggesting that expression of GHSR in vagal neurons is not sufficient for ghrelin-induced feeding. Ghrelin also fully increased food intake in AP-ablated mice (Cabral et al., 2017). Thus, the orexigenic effect of ghrelin in mice does not seem to require its action on the vagus nerve.

**Fig. 1:**
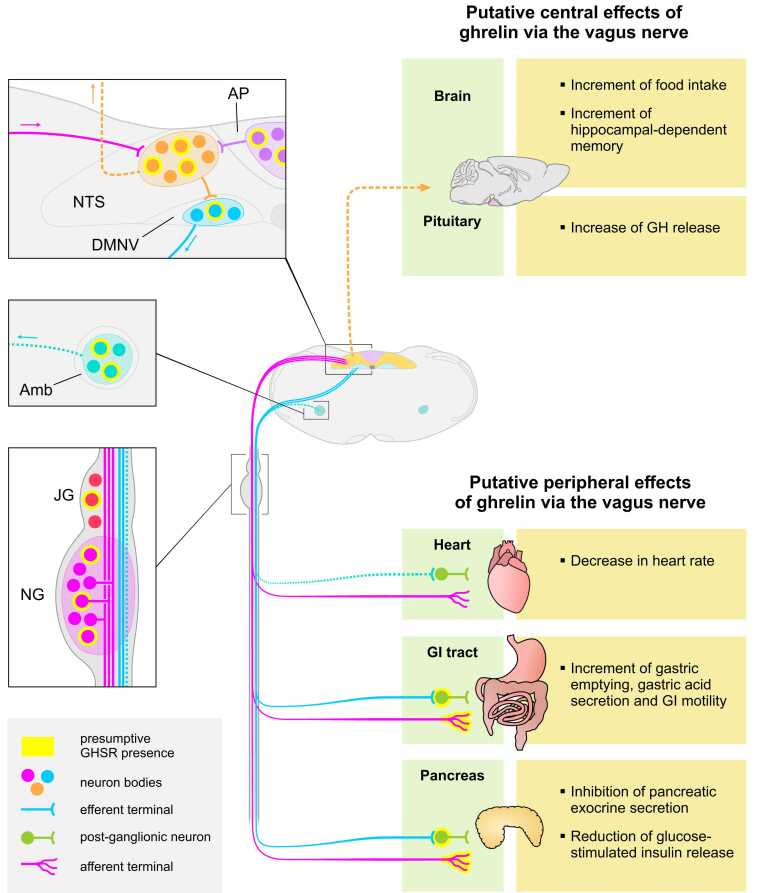
Schematic overview of the presumptive targets of ghrelin in the vagus nerve of rodents, and the putative actions of ghrelin mediated by the vagus nerve. The center of the figure depicts a coronal section of the medulla containing the DVC and Amb. Insets on the left show high magnification of the areas marked in the low magnification images. The DVC encompasses the NTS, the DMNV and the AP. The vagal efferent fibers (light blue) originate from the DMNV, responsible of the parasympathetic tone to visceral organs, including the GI tract and pancreas (solid light blue line), or the Amb, which generates branchial and parasympathetic motor efferents that innervate the heart (dotted light blue teal line). The depicted vagal afferents fibers (magenta) originate from pseudo-unipolar neurons of the NG, which transmit sensory information from specialized terminals located in different targets to the NTS. The NTS send projections (dotted orange line) that transmit vagal sensory information to brain regions that would mediate some ghrelin's actions. The AP senses circulating ghrelin and sends projections (purple line) to the NTS. Yellow borders around neurons or afferent terminals represent the presumptive presence of GHSR. Amb, nucleus ambiguus; AP, area postrema; DVC, dorsal vagal complex; DMNV, dorsal motor nucleus of the vagus; GHSR, growth hormone secretagogue receptor; JG, jugular ganglion; NG, nodose ganglion; NTS, nucleus of the solitary tract.

To our knowledge, only two studies investigated the orexigenic effect of ghrelin in patients with surgical procedures involving vagotomy. One study found that IV-injected ghrelin did not increase eating in seven patients with total subdiaphragmatic vagotomy (le Roux et al., 2005); however, this study did not include a control group of healthy individuals treated with ghrelin, raising concerns if the orexigenic effect of ghrelin could be unmasked in the tested experimental conditions. Another study found that ghrelin treatment increases food intake and appetite in ten patients with total gastrectomy (Adachi et al., 2010). Thus, the extent to which the vagus nerve is involved in the orexigenic effects of ghrelin in humans remains to be fully investigated. Fig. 1.

### 5.2 GH secretion

In contrast to ICV-injected ghrelin, IV-injected ghrelin was shown to be ineffective to induce GH release 7 days after total subdiaphragmatic vagotomy or gastric branch vagotomy in rats (Date et al., 2002). Similarly, a subsequent study found that IV- or ICV-injected ghrelin display an attenuated effect on plasma GH secretion in rats 7 days after total subdiaphragmatic vagotomy, which in turn reduced basal plasma levels of GH and insulin-like growth factor 1 as well as GHSR gene expression in the pituitary (Al-Massadi et al., 2011). The notion that ghrelin-induced GH-release involves a vagus-mediated pathway seems surprising, particularly in light of the observation that ghrelin potently increases GH secretion from primary cultures of rat pituitary cells (Kojima et al., 1999). *In vivo*, SC-injected ghrelin mainly increases GH secretion by acting on the pituitary since ghrelin did not induce GH secretion in mice with specific deletion of GHSR in somatotropic cells (Gupta et al., 2021). It seems likely that the effect of ghrelin on GH release in vagotomized rats tested few days after surgery is affected by stress, which potently reduces GH secretion in rodents (Xu et al., 2011). To our knowledge, ghrelin-induced GH release was not investigated in vagotomized mice, but IP-injected ghrelin did not increase GH release in mice with selective GHSR expression in Phox2b-expressing neurons (Okada et al., 2018). Importantly, IV-injected ghrelin induced GH release in vagotomized patients, as seen in healthy subjects (le Roux et al., 2005, Takeno et al., 2004). Thus, studies in mice and humans indicate that ghrelin-induced release of GH does not require its action on the vagus nerve.

### 5.3 Gastrointestinal tract functions

Most studies investigating the role of the vagus nerve mediating ghrelin’s effects on GI tract functions were reported in rats. An early study showed that IV-injected ghrelin displays attenuated stimulatory effects on GI motility and gastric acid secretion in rats either pretreated with atropine to block cholinergic receptors or subjected to cervical vagotomy (Masuda et al., 2000). Similarly, ICV-injected ghrelin did not increase gastric acid secretion in rats with gastric vagotomy or treated with atropine (Date et al., 2001). Further studies in rats confirmed that vagal function integrity is required for the stimulatory effects of systemically-injected ghrelin on gastric acid secretion (Fukumoto et al., 2008, Sakurada et al., 2010) and gastric emptying (Fukuda et al., 2004). Furthermore, GHSR knockdown in the NG slowed gastric emptying in rats (Davis et al., 2020). *Ex vivo* recordings showed that ghrelin directly modulates vagal afferents of the GI tract in anaesthetized rats. In particular, IV-injected ghrelin suppressed gastric vagal afferent activity (Date et al., 2005, Date et al., 2002, Zhang et al., 2020), and ghrelin hyperpolarized ~30% of duodenum-projecting NG neurons, through a mechanism involving potassium channels (Grabauskas et al., 2015). Conversely, intra-gastric administration of a GHSR antagonist or a GHSR inverse agonist dose-dependently increased gastric vagal afferents activity (Kong et al., 2016). Recently, IV-injected ghrelin was shown to decrease gastric mechanosensitivity in response to graded distensions in rats, and such effect was blocked by total subdiaphragmatic vagotomy (Meleine et al., 2020). Ghrelin-mediated suppression of vagal afferent activity could contribute to increase food intake, since stomach distention inhibits feeding via stimulation of vagal mechano-sensory afferents, and to decrease the inhibitory vagovagal-mediated effects on gastric emptying and GI motility (Page et al., 2007). Strikingly, the study by Arnold and colleagues, which was unable to confirm that ghrelin-induced food intake requires the integrity of the vagus nerve, found that intra-carotid ghrelin neither affected spontaneous nor stimulated activity of load-sensitive vagal afferents (Arnold et al., 2006). Also, ghrelin was shown to increase, rather than decrease, vagal activity in an *in situ* model of decorticated artificially perfused rats, an effect that was blunted by GHSR knockdown in the NG (Davis et al., 2020). Thus, some discrepancies seem to exist regarding to the action of ghrelin on the activity of vagal afferents. On top of its putative effect on vagal afferents, ICV-injected ghrelin increases efferents activity of the posterior subdiaphragmatic vagal trunk in anesthetized rats (Sato et al., 2003), and intra-DVC injected ghrelin elicited gastric motility through excitation of the preganglionic motor neurons of the DMNV that, in turn, activate postganglionic cholinergic neurons (Swartz et al., 2014). Overall, evidence indicates that ghrelin has a potent effect on gastric emptying and gastric acid secretion in rats that may involve integrated effects on afferent and efferent fibers of the vagus nerve.

Few studies explored the effect of ghrelin on GI tract functions in mice. In an electrophysiological set up, IV-injected ghrelin suppressed gastric vagal afferent activity in mice (Asakawa et al., 2001). In *ex vivo* mouse gastro-esophageal preparations, ghrelin reduced both spontaneous and stimulated response of vagal gastro-esophageal afferents that respond to tension receptors (Kentish et al., 2012, Page et al., 2007). Thus, ghrelin seems to inhibit vagal afferents in mice, but the implications of this action remain to be studied. In terms of the effects of ghrelin on GI function, it is important to highlight that mice and rats lack the GI-derived hormone motilin and its receptor, which play key roles regulating GI motility and gastric secretion in other species, including in humans (Kitazawa and Kaiya, 2019). Based on such important limitation of mice and rats as a model to study the role of ghrelin on the GI tract of humans, many studies have used other species, such as ferrets, guinea pig and Asian house shrew, among others. The role of the vagus nerve mediating ghrelin actions on GI motility on different animal models has been recently reviewed, and most studies indicated that ghrelin regulates GI motility, at least in part, via vagal mechanisms in most mammals (Kitazawa and Kaiya, 2019).

The prokinetic activity of ghrelin on the GI tract also involves the enteric nervous system, which controls the migrating motor complex (MMC). The MMC is a pattern of electrical activity detected in the GI tract during fasting and is usually divided in three phases. Ghrelin treatment was shown to induce premature phase III contractions in rats (Fujino et al., 2003), mice (Zheng et al., 2009) and humans (Tack et al., 2006). In rats, plasma ghrelin levels was shown to peak before the onset of phase-III contractions (Ariga et al., 2007, Taniguchi et al., 2008), and the administration of a GHSR antagonist reduced the number of phase-III contractions (Ariga et al., 2007). Some evidence indicates that the action of ghrelin on the MMC involves vagal efferent signals, which are known to disrupt the MMC at the gastric level (Deloose et al., 2012). For instance, ICV-injected ghrelin did not increase MMC activity in the duodenum or gastric antrum of rats with truncal vagotomy (Fujino et al., 2003). However, IV-injected ghrelin increased duodenal motility in rats with truncal vagotomy, suggesting that plasma ghrelin may also act directly on the GI tract to regulate motor activity (Fujino et al., 2003). In line with such notion, GHSR-immunoreactivity was detected in the intrinsic neurons of the enteric nerve plexuses but absent in smooth muscle cells and epithelia of rat and human stomach and colon (Dass et al., 2003). In rat isolated forestomach circular muscle, ghrelin dose-dependently facilitated the nerve stimulation-evoked contractions (Dass et al., 2003). Also, ghrelin stimulates isometric contractions of segments of the rat jejunum *in vitro* in a cholinergic dependent-manner and dose-dependently shortened the MMC cycle length in rat duodenum and jejunum (Edholm et al., 2004). Similarly, ghrelin enhanced electrical field stimulation-induced contractions of strips of the gastric body (Fukuda et al., 2004).

To our knowledge, only one study investigated the requirement of the vagus nerve for ghrelin’s effects on GI tract functions in humans and found that ghrelin increases gastric emptying in one patient with neurogenic gastroparesis due to truncal vagotomy, suggesting that ghrelin can exert prokinetic actions on the stomach via extra-vagal mechanisms (Binn et al., 2006).

### 5.4 Pancreas

An early study reported that IV-injected ghrelin inhibits pancreatic protein secretion induced by either central vagal stimulation or a muscarinic receptor agonist in rats (Zhang et al., 2001). The same study showed that IV-injected ghrelin inhibits CCK-induced pancreatic secretion in rats with total subdiaphragmatic vagotomy and that ghrelin does not act on dispersed pancreatic acini, suggesting that ghrelin inhibits pancreatic secretion regulating intrapancreatic neurotransmission (Zhang et al., 2001). A further study showed that ICV-, but not IV-, injected ghrelin in rats dose-dependently increased pancreatic exocrine secretion, and also that the pretreatment with a nicotinic acetylcholine receptor antagonist or with atropine abolished the stimulatory effect of ICV-injected ghrelin, suggesting that central action of ghrelin can activate vagal efferents to reduce pancreatic secretion (Sato et al., 2003). Another study found that the effect of IV-injected ghrelin on pancreatic protein secretion is impaired in rats with total subdiaphragmatic vagotomy or pretreated with atropine, but not in rats that had received perivagal capsaicin treatment at the abdominal vagal trunks (Li et al., 2006). The same study showed that ghrelin does not increase pancreatic protein secretion in AP-ablated rats (Li et al., 2006) and concludes that plasma ghrelin stimulates pancreatic secretion via a cholinergic efferent vagal pathway that involves the AP. Thus, several studies have shown that ghrelin inhibits pancreatic secretion in rats via an autonomic mechanism, but the intricacies of the neuronal circuit by which ghrelin acts need to be further clarified.

In humans, two studies reported that IV-injected ghrelin increases pancreatic polypeptide secretion, which could be indicative of an effect of ghrelin on vagal afferents. Specifically, one study showed that ghrelin increases pancreatic polypeptide secretion in individuals with obesity, but not in lean neither in obese post-RYGB subjects (Tamboli et al., 2017), whereas another study found that ghrelin-induced increase in pancreatic polypeptide is blunted by alcohol administration (Farokhnia et al., 2021).

The vagus nerve may also mediate some actions of ghrelin on the endocrine pancreas. In rats, ghrelin infused into the portal vein, but not in the femoral vein, inhibited glucose-stimulated insulin release, and hepatic vagotomy or atropine pretreatment reduces such effect of ghrelin, suggesting that it involves the vagus nerve (Cui et al., 2008). Notably, IP-injected ghrelin increased glycemia in mice with GHSR expression restricted to Phox2b-expressing neurons in a similar extent as seen in wild-type mice, but not in GHSR-deficient mice, suggesting that the action of ghrelin in Phox2b-expressing neurons is sufficient to induce its acute hyperglycemic effects (Okada et al., 2018). GHSR knockdown in the NG of rats leaded to higher hyperglycemia after glucose infusion and increased postprandial insulin levels (Davis et al., 2020). Thus, ghrelin seems to directly act on vagal afferents and efferents to modulate the function of the endocrine pancreas in rats and mice.

### 5.5 Cardiovascular system

The first indication that ghrelin affects the cardiovascular system via the vagus nerve came from the finding that intra-NTS-, but not intra-AP-, injected ghrelin dose-dependently decreased heart rate and mean arterial pressure in rats and that atropine blocked such effect (Lin et al., 2004). Analysis of heart-rate variability revealed that IV-injected ghrelin increases parasympathetic activity and decreases sympathetic activity in rats, and that these effects of ghrelin were impaired by atropine pretreatment or capsaicin-induced cardiac vagotomy (Soeki et al., 2013). In mice, SC-injected ghrelin inhibited cardiac sympathetic nerve activity, reduced malignant arrhythmia and improved prognosis after myocardial infarction (Mao et al., 2012). Due to the major differences between rodent and human hearts, rabbit has been used as an alternative model to study the role of ghrelin on the cardiovascular system. In this regard, ICV-injected ghrelin in conscious rabbits dose-dependently decreased arterial pressure and heart rate (Matsumura et al., 2002), and such effect correlated with acetylcholine release into the right atrium and was impaired after transection of cervical vagal nerves (Shimizu et al., 2011). In healthy individuals, IV-injected ghrelin inhibited cardiac sympathetic activity with a moderate effect on cardiac parasympathetic activity, whereas it has no effect in vagotomized subjects (Huda et al., 2010). Also, Holter electrocardiography recordings in humans showed that ghrelin suppresses cardiac sympathetic activity and stimulates cardiac parasympathetic activity (Soeki et al., 2013). Thus, the effects of ghrelin on the cardiovascular system appear to be strongly mediated through regulation of the autonomic nervous system.

## 6 Concluding remarks and future directions

Here, we attempted to provide a compelling summary of the current evidence that supports or refutes the notion that some ghrelin's actions involve the vagus nerve and tried to identify some key controversies in the topic (Table 1). Based on the discussed information, we conclude that the use of rats as an experimental model provided strong evidence indicating that the vagus nerve is required for some actions of ghrelin on the digestive and cardiovascular systems. Conversely, the putative role of the vagus nerve mediating the orexigenic and GH releasing properties of ghrelin remains uncertain. Importantly, most of the studies in rats were performed in vagotomized animals, and consequently, allow to infer if a given effect of ghrelin requires, or not, the integrity of the vagus nerve but preclude to determine if ghrelin directly acts on vagal neurons, as occasionally erroneously concluded in some articles. Furthermore, we conclude that it is still controversial if mouse vagal sensory neurons express GHSR. Thus, the use of genetically manipulated mice to investigate the role of the vagus nerve in mediating some ghrelin's actions is currently under question. Finally, we noticed that the role of the vagus nerve mediating ghrelin's actions in humans was investigated in few studies, which, in turn, usually had low sample sizes and/or lack accurate and standardized protocols. Thus, studies in humans are needed to clarify the extent to which observations in animal models can be extrapolated to the human physiology.

**Table 1:** Summary of the arguments in favor or against some key controversial aspects of the putative role of the vagus nerve mediating some actions of ghrelin.

Controversial notion	Key observations in favor	Key observations against
Ghrelin-induced food intake in humans involves the vagus nerve.	-IV-injected ghrelin does not increase eating in 7 patients with total subdiaphragmatic vagotomy (le Roux et al., 2005).	-IV-injected ghrelin increases food intake and appetite in ten patients with total gastrectomy (Adachi et al., 2010).
Ghrelin-induced food intake in rats involves the sensory neurons of the vagus nerve.	-IV-injected ghrelin does not increase food intake in rats 7 days after total subdiaphragmatic vagotomy or in rats with capsaicin-induced or surgical gastric vagotomy (Date et al., 2002). -IP-injected ghrelin does not increase food intake in 16-h food deprived rats receiving perivagal treatment with capsaicin at the cervical level (Chen et al., 2005). - IP-injected ghrelin does not increase food intake during the nocturnal phase in rats tested ~3 months after a total subdiaphragmatic vagotomy (Davis et al., 2020).	-IP-injected ghrelin fully increases food intake in rats with either subdiaphragmatic vagal deafferentation or total subdiaphragmatic vagotomy (Arnold et al., 2006).
Ghrelin-induced GH release in rats involves the vagus nerve.	-IV-injected ghrelin does not increase GH release in rats 7 days after total subdiaphragmatic vagotomy or in rats with capsaicin-induced or surgical gastric vagotomy (Date et al., 2002). -IV- or ICV-injected ghrelin display an attenuated effect on plasma GH secretion in rats 7 days after total subdiaphragmatic vagotomy (Al-Massadi et al., 2011).	-A great body of evidence indicates that ghrelin controls GH secretion in rats acting at hypothalamic and pituitary levels (Steyn et al., 2016).
GHSR is expressed in sensory neurons of the vagus nerve of mice.	-Widespread GHSR was detected in the mouse NG using IHC, ISH, fluorescent ISH, reverse transcription PCR (Christie et al., 2020, Davis et al., 2020, Kentish et al., 2012, Page et al., 2007, Zhang et al., 2020).	-GHSR mRNA undetectable or detected in a small subset of JG neurons in mice using qPCR, single-cell RNA sequencing or multiplex ISH (Bob-Manuel and Gautron, 2021, Egerod et al., 2018, Kupari et al., 2019).

Future research on the role of the vagus nerve mediating the actions of ghrelin should not only make use of state-of-the-art technologies (such as optogenetics or pharmacogenetics) but also take in consideration the most recent findings in the field. For instance, GHSR signaling is known to modulate reward-related behaviors and memory consolidation (Cornejo et al., 2021a), and recent evidence shows that the vagus nerve also affects such processes (Han et al., 2018, O’Leary et al., 2018, Suarez et al., 2018, Tellez et al., 2013). Thus, it can be hypothesized that the vagus nerve mediates, at least in part, the action of ghrelin on reward and memory formation. In line with such possibility, GHSR knockdown in the NG of rats was shown to impair hippocampal-dependent memory and to reduce brain-derived neurotrophic factor levels in the hippocampus, but did not affect appetitive contextual or spatial memory (Davis et al., 2020). It is likely that future studies will further help to gain more insights about such exciting notions. Also, it is interesting to stress that the liver-expressed antimicrobial peptide 2 (LEAP2) was recently identified as a novel GHSR ligand that blocks ghrelin-evoked and constitutive GHSR activities (Ge et al., 2018, M’Kadmi et al., 2019). Plasma LEAP2 levels display an inverse relationship to plasma ghrelin levels: increasing in fed states and decreasing in fasting states (Cornejo et al., 2021b). Notably, LEAP2 is produced in the liver and jejunum, and consequently it could directly impact on the putative GHSR-expressing terminals of the vagal fibers. Thus, the study of the role of LEAP2 on vagal fibers is another aspect of GHSR signaling on the vagus nerve activity that may bring novel concepts to the field.

## CRediT authorship contribution statement

**Mario Perelló:** Writing – original draft preparation, Writing – review & editing. **María P Cornejo:** Writing – review & editing. **Pablo N De Francesco:** Writing – review & editing. **Gimena Fernandez:** Writing – review & editing. **Laurent Gautron:** Writing – review & editing. **Lesly S Valdivia:** Writing – review & editing.
